# A Sample-to-Report Solution for Taxonomic Identification of Cultured Bacteria in the Clinical Setting Based on Nanopore Sequencing

**DOI:** 10.1128/JCM.00060-20

**Published:** 2020-05-26

**Authors:** Stefan Moritz Neuenschwander, Miguel Angel Terrazos Miani, Heiko Amlang, Carmen Perroulaz, Pascal Bittel, Carlo Casanova, Sara Droz, Jean-Pierre Flandrois, Stephen L. Leib, Franziska Suter-Riniker, Alban Ramette

**Affiliations:** a University of Bern, Institute for Infectious Diseases, Bern, Switzerland; b University of Lyon, CNRS, UMR 5558, Laboratoire de Biométrie et Biologie Evolutive, Villeurbanne, France; Johns Hopkins University School of Medicine

**Keywords:** taxonomy, 16S RNA gene, bioinformatics, clinical methods, diagnostics, nanopore, sequencing

## Abstract

Amplicon sequencing of the 16S rRNA gene is commonly used for the identification of bacterial isolates in diagnostic laboratories and mostly relies on the Sanger sequencing method. The latter, however, suffers from a number of limitations, with the most significant being the inability to resolve mixed amplicons when closely related species are coamplified from a mixed culture. This often leads to either increased turnaround time or absence of usable sequence data. Short-read next-generation sequencing (NGS) technologies could solve the mixed amplicon issue but would lack both cost efficiency at low throughput and fast turnaround times. Nanopore sequencing developed by Oxford Nanopore Technologies (ONT) could solve those issues by enabling a flexible number of samples per run and an adjustable sequencing time. Here, we report on the development of a standardized laboratory workflow combined with a fully automated analysis pipeline *LORCAN* (long read consensus analysis), which together provide a sample-to-report solution for amplicon sequencing and taxonomic identification of the resulting consensus sequences. Validation of the approach was conducted on a panel of reference strains and on clinical samples consisting of single or mixed rRNA amplicons associated with various bacterial genera by direct comparison to the corresponding Sanger sequences. Additionally, simulated read and amplicon mixtures were used to assess *LORCAN*’s behavior when dealing with samples with known cross-contamination levels. We demonstrate that by combining ONT amplicon sequencing results with *LORCAN*, the accuracy of Sanger sequencing can be closely matched (>99.6% sequence identity) and that mixed samples can be resolved at the single-base resolution level. The presented approach has the potential to significantly improve the flexibility, reliability, and availability of amplicon sequencing in diagnostic settings.

## Abstract (precis)

Amplicon sequencing of the 16S rRNA gene is commonly used for the identification of bacterial isolates in diagnostic laboratories and mostly relies on the Sanger sequencing method. The latter, however, suffers from a number of limitations, with the most significant being the inability to resolve mixed amplicons when closely related species are coamplified from a mixed culture. This often leads to either increased turnaround time or absence of usable sequence data. Short-read next-generation sequencing (NGS) technologies could solve the mixed amplicon issue but would lack both cost efficiency at low throughput and fast turnaround times.

## INTRODUCTION

The sequencing of the 16S rRNA gene is essential to describe the diversity of the human microbiome (1, 2). Yet, the frequency of the use of 16S sequencing for species identification from cultured isolates in clinical laboratories is decreasing (3) despite the usefulness of 16S rRNA gene sequencing to provide taxonomic classification for isolates that do not match recognized biochemical profiles, that only produce low identification score according to commercial systems, or that are not typically associated with human pathogens (3, 4). In the clinical microbiology laboratory, amplicon sequencing of the 16S rRNA gene mostly relies on the Sanger sequencing method, which is based on chain termination via fluorescently labeled deoxyribonucleotides (dNTPs), capillary electrophoresis, and fluorescence measurement (5). Although the Sanger method is still the gold standard for validating the accuracy of sequences from specific genes, when compared to more recent technologies, the method has a number of significant shortcomings. During a sequencing run, each capillary is limited to the production of one single sequence with a maximal length of about 1,000 bp (6), resulting in low throughput and high sequencing costs. Furthermore, the sequencing machines are comparably large and require maintenance, limiting their suitability for all types of laboratory settings. The most important limitation of the Sanger method, however, is its limited ability to produce complete sequence information when diverse amplicons are present (7). Under routine diagnostic conditions, this frequently leads to either increased turnaround time or lack of results (8), leading to potential delays or inaccuracies in patient treatment and management.

Next-generation sequencing technologies (i.e., second-generation sequencing technologies, such as those provided by Illumina) might overcome most of these limitations but are not designed for the analysis of small numbers of pure amplicons. Even the smallest and fastest available 500- and 600-cycle Illumina kits show runtimes of >24 h, with associated running costs of several hundred euro regardless of the numbers of samples processed (Illumina, Inc.), limiting their usefulness for the fast and flexible identification of small batches of samples. The third-generation single-molecule sequencing technology provided by Oxford Nanopore Technologies (ONT) might offer the necessary flexibility in throughput and is capable of producing reads with lengths of several hundred to several hundred thousand bases at competitive costs (9). Furthermore, ONT sequencers are small devices, virtually maintenance free, and affordable for small laboratories. Despite the constant improvement over the last years in read accuracy (with read accuracy of about 96% currently), the remaining sequencing errors in single nanopore reads do not yet allow for an analysis at the read level. *De novo* assembly or consensus generation from individual ONT reads are, therefore, commonly used to generate sequences that are virtually free from substitution errors (10). Additionally, “polishing” tools can be applied to remove remaining nonrandom errors, such as indels in homopolymer regions, from the generated consensus sequences (10–13). Resulting sequences can then be directly substituted to Sanger sequences in existing classification pipelines or, due to the added flexibility in read length, may provide far higher resolution if the analyses are based on full-length marker genes or entire operons (14). One obstacle for a broad adoption of nanopore sequencing in routine diagnostic laboratories is the added bioinformatic complexity compared to established Sanger sequencing workflows. Furthermore, available workflows are often limited to the analysis of pure amplicons (10–13), include complex modifications of the ONT laboratory workflows (15, 16), or lack published validation by using samples other than mock communities (17, 18).

Here, we developed a complete workflow based on standard ONT protocols and a fully automated analysis pipeline *LORCAN* capable of producing high-quality consensus sequences and thorough taxonomic analysis from pure and low-complexity cultures. The foreseen end users of the workflow are clinical bacteriology laboratories. As such, tunable workflow parameters were evaluated with amplicons generated from reference strains of pathogenic genera (*Bacteroides*, *Eggerthella*, *Enterococcus*, *Klebsiella*, *Mycobacterium*, *Campylobacter*, *Pseudomonas*) and validated on bacterial cultures obtained from patient material over several months. Furthermore, we explored the robustness of *LORCAN*’s consensus generation and species identification by analyzing artificial mixtures of amplicons and reads at different levels of genetic distances.

## MATERIALS AND METHODS

### Samples, DNA extraction, PCR amplification.

Bacterial isolates all originated from the Institute for Infectious Diseases (IFIK, Bern) Biobank. The IFIK provides the entire spectrum of medical microbiological diagnostic services to the largest Swiss hospital group (Inselgruppe) and other regional hospitals. The diagnostic division of IFIK (clinical microbiology) is ISO/IEC 17025 accredited to perform routine bacterial diagnostics from clinical samples. ATCC strains were obtained from LGC Standards (Wesel, Germany) and were grown on solid medium as recommended by the manufacturer.

Bacterial cultures grown overnight were harvested from agar plates and dissolved in 300 μl of Tris-EDTA (pH 8.0). DNA was extracted with a NucliSens easyMag (bioMérieux, Switzerland) robot according to the manufacturer’s protocol. The 16S rRNA gene PCR was performed with the primer sets 16S_f, 5′-AGAGTTTGATCMTGGCTCAG-3′, and 16S_r, 5′-TACCGCGGCWGCTGGCACRDA-3′, (general bacteria) and mbak_f, 5′-GAGTTTGATCCTGGCTCAGGA-3′, and mbak_r, 5′-TGCACACAGGCCACAAGGGA-3′, (mycobacteria) supplemented with the universal tails 5′-TTTCTGTTGGTGCTGATATTGC-3′ (ONT forward primer), 5′-ACTTGCCTGTCGCTCTATCTTC-3′ (ONT reverse primer), 5′-TGTAAAACGACGGCCAG-3′ (M13f, Sanger forward primer), or 5′-CAGGAAACAGCTATGAC-3′ (M13r, Sanger reverse primer). PCR mixtures (25 μl) for general bacteria and mycobacteria were assembled, respectively, with 1 and 2.5 ng DNA template and 10 μl of a 1.25 and 2.5 μM primer working solution, both with 12.5 μl Q5 master mix. Amplification was performed in a GeneAmp 9700 thermocycler (Thermo Fisher Scientific, Inc., MA, USA) with the following program: 98°C for 1 min; 30 cycles of 98°C for 10 s, 63°C for 15 s, 72°C for 30 s; and 72°C for 2 min. PCR products were purified with CleanNGS beads (CleanNA, Waddinxveen, Netherlands) according to the manufacturer’s instructions with the following modifications: after the washing step, an additional 3-s centrifugation step was introduced, and the purified DNA was eluted in 80 μl of Tris-HCl (0.01 M, pH 8.0). Fragment size of the amplicons was analyzed using the TapeStation D1000 assay (Agilent, Santa Clara, CA, USA), concentrations were measured with the Qubit double-stranded DNA (dsDNA) broad-range (BR) assay (Thermo Fisher Scientific), and the purity of the DNA was analyzed with a NanoDrop spectrophotometer (Thermo Fisher Scientific). Samples with DNA concentrations of <1.05 nM were excluded from the analysis.

### Library preparation.

**FIG 1:**
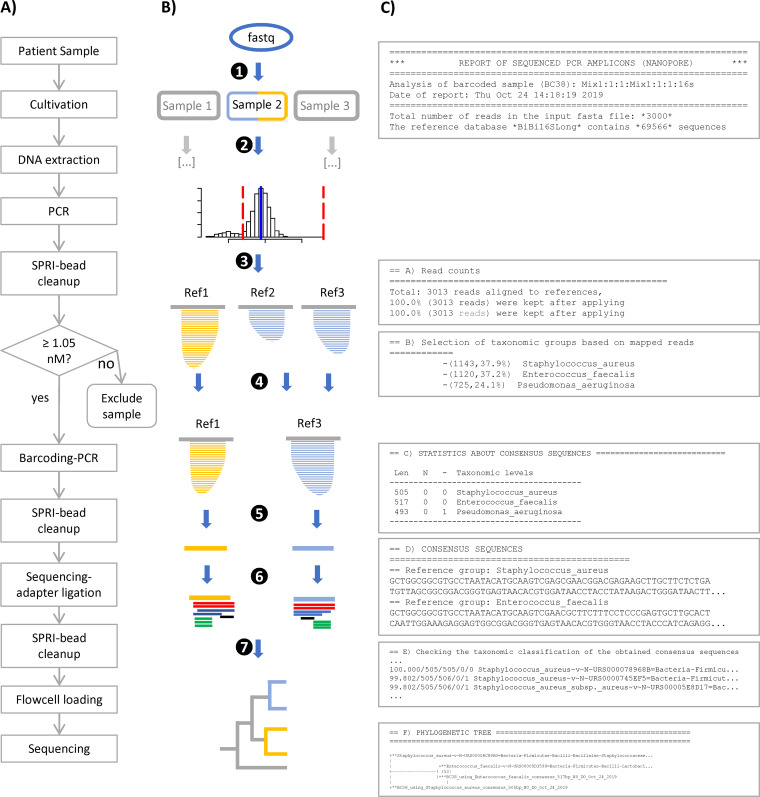
(A) Overview of the wet laboratory workflow. Steps of the *LORCAN* analysis (B) and corresponding sections of the generated report (C). (Step 1) Demultiplexing and adapter trimming. (Step 2) Read filtering by size. (Step 3) Mapping to a reference database. (Step 4) Read extraction, binning by species, and remapping. (Step 5) Consensus calling. (Step 6) Selection of the closest references by BLAST. (Step 7) Taxonomic tree building.

A typical library consisted of the pooling of PCR amplicons from 2 to 15 clinical samples and 1 positive control (Mycobacteria intracellulare, amplified with general bacterial primers). Library preparation was performed with the kits EXP-PBC096 and SQL-LSK109 (Oxford Nanopore Technologies, Oxford, UK) using the supplementary reagents NEBNext end repair/dA-tailing module (E7546; New England Biolabs, ON, CA), NEB Blunt/TA ligase master mix (M0367; New England Biolabs), *Taq* 2× master mix (NEB M0270; New England Biolabs), and CleanNGS beads (CleanNA). All modifications made to the manufacturer’s protocol (PCR barcoding [96] genomic DNA, PBAC96_9069_v109_revK_14Aug2019) are described in the following section (see also Fig. 1A; for a detailed protocol, see Text S1 in the supplemental material). AMPure beads were substituted with CleanNGS beads, and the HulaMixer (Thermo Fisher Scientific) parameters “orbital: 40 rpm, 07 s; reciprocal: 89 deg, 2 s; vibro: 5 deg, 2 s; vertical position” were used. Barcoding PCRs (12 cycles) were set up with 25.2 nmol of template per reaction. Raw barcoded PCR products were quantified with the Qubit dsDNA BR assay and pooled at equal molar proportions. Products containing less than 0.57 pmol DNA were excluded from the analysis. If the total amount of DNA in a pooled library was below 9.23 pmol, “place-holder” (filling) barcoded samples were added to the pooled library to avoid flow cell underloading (see example of calculations and adjustments in Text S1). Place-holder barcoded samples were produced in advance from the same template as the positive controls, with 15 instead of 12 barcoding PCR cycles. Resulting PCR products were quantified with Qubit and stored at −20°C. The pooled library was purified (CleanNGS beads; 50-μl elution volume) and quantified with the Qubit dsDNA BR assay. The purified library pools were diluted to 140 nM before proceeding to the “end preparation” step of the protocol.

### Sequencing.

ONT sequencing was performed on a GridION X5 instrument (Oxford Nanopore Technologies) with real-time basecalling enabled (*ont-guppy-for-gridion* v.1.4.3-1 and v.3.0.3-1; fast basecalling mode). Sequencing runs were terminated after production of 1 million reads or when sequencing rates dropped below 20 reads per second. Purified PCR products were submitted to Sanger sequencing at Microsynth (Balgach, Switzerland).

### Bioinformatic analyses.

**(i) *LORCAN* pipeline description.** *LORCAN* was developed to facilitate reproducible ONT sequencing-based marker gene analysis in diagnostics facilities. The pipeline, written in Perl 5, R, and BASH, runs on Linux servers or workstations. The code is publicly available (19) and is based on publicly available, third-party dependencies (see Table S1 in the supplemental material). Major steps of the workflow are described in the following section (numbers correspond to the steps in Fig. 1B). In step 1, basecalled reads are demultiplexed and adapters trimmed (Porechop [20], parameters: –format fasta, –discard_unassigned, –require_two_barcodes). In step 2, reads are filtered by length, keeping only those with lengths of −20 to +100 bases (lower boundary adjustable) around the modal sequence length (custom Perl and R scripts) (Fig. 1B). In step 3, reads are mapped to a nonredundant reference database (minimap2 [21]; see database preparation below). In step 4, reads are extracted, binned by taxonomic level (here species), and remapped to the reference sequence that obtained the highest number of mapped reads among all sequences of the corresponding species (minimap2, SAMtools [22], SeqKit [23]). In step 5, consensus sequences are derived using a 50% majority rule consensus. In step 6, the 10 closest reference sequences are selected by sequence similarity to the consensus sequence (blastn, BLAST+ [24]). In step 7, phylogenetic trees for each consensus sequence with its 10 closest references are created (MAFFT [25] with parameters -*maxiterate 1000 –localpair*; gBlocks [26] with parameters *−t = d*; and IQ-Tree [27] with parameters -*m GTR+I+G -bb 1000 -czb*). Parameters of all software are also provided in the *LORCAN* GitHub repository.

**(ii) Database preparation.** Reference databases used by *LORCAN* are nonredundant and assay specific. Detailed instructions for database creation are provided online (https://github.com/aramette/LORCAN/). In short, the reference database (in this study, leBIBI SSU-rDNA-mk37_stringent, https://umr5558-bibiserv.univ-lyon1.fr/BIBIDOCNEW/db-BIBI.html [28]) was trimmed to the region of interest (amplified region minus primers) and dereplicated (mothur [29]), and sequence names were simplified (custom Perl scripts). The names of identical sequences are saved to a file during the dereplication step. The resulting nonredundant database is then used to generate a custom BLAST database, which is used in *LORCAN* pipeline.

**(iii) Sanger sequence analyses.** Forward and reverse sequences were assembled into consensus sequences using SeqMan Pro (DNA Star, Madison, WI, USA), primers were trimmed manually, and ambiguous bases were resolved based on visual inspection of the chromatograms. Consensus sequences were taxonomically classified using the online tool leBIBI QBPP (28, 30).

**(iv) SNV discrimination and performance with mixed samples.** Amplicons produced from pure samples were quantified (Qubit dsDNA BR assay). Mixtures of pure amplicons were produced at defined ratios before library preparation to produce libraries of heterogeneous (“mixed”) samples. Artificial read mixtures were also produced *in silico* by mixing reads originating from pure amplicon samples. Those reads were obtained from the *LORCAN* output directories (output file 1_fasta/BC*.fasta produced by step 2) (Fig. 1B) and sampled using Seqtk subseq (v.1.3-r106) (https://github.com/lh3/seqtk) to produce different proportions of original, pure amplicons. Reads from mixed amplicon samples were fed back into *LORCAN*, and detected species compositions were extracted from the resulting *LORCAN* reports. Sequence identities between the paired *Mycobacterium* species were determined based on pairwise alignment of the amplified region using MultAlin v.5.4.1 (http://multalin.toulouse.inra.fr/multalin/ [31]).

**(v) Influence of database completeness on consensus accuracy.** Amplicons from a set of seven ATCC reference strains were ONT sequenced and analyzed with *LORCAN* using the full nonredundant leBIBI 16S rRNA database, restricted to the region amplified by the general bacterial primer set. The resulting top consensus sequences were extracted and combined with the above-mentioned database. The resulting sequence data set was aligned (MAFFT v.7.313, FFT-NS-1, progressive method), and pairwise distances were calculated (mothur v.1.40.5, *dist.seqs*, calc = eachgap, countends = F, cutoff = 0.20). For each consensus sequence, 10 subsets of sequences with minimal distances below thresholds ranging from 0 to 0.1 were extracted (Seqtk subseq), and minimal distances between each data set and the corresponding consensus sequence were analyzed. The seven ATCC read sets were reanalyzed with *LORCAN* and the corresponding database subsets to produce consensus sequences. Top consensus sequences from each combination of sample and subsetted database were extracted, combined with the consensus sequences generated with the full database, and aligned (MAFFT v.7.313, L-INS-I, iterative refinement method (<16) with local pairwise alignment information). Pairwise distances were analyzed as described above, and distances between the consensus sequences generated with the full and the subsetted databases were extracted.

### Data availability.

All reads and consensus sequences corresponding to the data presented in Table 1 and the *LORCAN*-derived consensus sequences used as references in Fig. 3 were deposited in the European Nucleotide Archive under project accession number PRJEB34167 or made available as supplementary multi-FASTA files.

**TABLE 1:** Validation of taxonomic classification of ATCC reference strains^*a*^

ATCC strain reference no.	Taxonomy	*LORCAN* top consensus sequence		SANGER consensus sequence leBIBI QBPP taxonomy^*b*^	*LORCAN* vs Sanger consensus sequence identity (%)
		*LORCAN* taxonomy	leBIBI QBPP taxonomy^*b*^		
33560	Campylobacter jejuni subsp. *jejuni*	Campylobacter jejuni	[Campylobacter lari subsp. *concheus*, Campylobacter jejuni subsp. jejuni*, Campylobacter jejuni subsp. *doylei*] (and 2 others)	[Campylobacter lari subsp. *concheus*, Campylobacter jejuni subsp. jejuni*, Campylobacter jejuni subsp. *doylei*] (and 2 others)	99.77
43504	Helicobacter pylori	Helicobacter pylori	[Helicobacter pylori*]	[Helicobacter pylori*]	99.54
29212	Enterococcus faecalis	Enterococcus faecalis	[Enterococcus faecalis*]	[Enterococcus faecalis*]	100.00
25922	Escherichia coli	Escherichia coli	[Escherichia marmotae, Escherichia fergusonii] Shigella flexneri*	[Shigella flexneri]	99.57
49247	Haemophilus influenzae	Haemophilus influenzae	[Haemophilus influenzae*]	[Haemophilus influenzae*]	98.94
49226	Neisseria gonorrhoeae	Neisseria gonorrhoeae	[Neisseria gonorrhoeae*]	[Neisseria gonorrhoeae*]	100.00
27853	Pseudomonas aeruginosa	Pseudomonas aeruginosa	[Pseudomonas tropicalis*, Pseudomonas aeruginosa, Pseudomonas hussainii]	[Pseudomonas tropicalis*, Pseudomonas indica, Pseudomonas aeruginosa]	99.78
25923	Staphylococcus aureus	Staphylococcus aureus	[Staphylococcus aureus subsp. anaerobius*]	[Staphylococcus argenteus, Staphylococcus aureus subsp. *aureus*, Staphylococcus schweitzeri*] (and 2 others)	99.79
49619	Streptococcus pneumoniae	Streptococcus pneumoniae	[Streptococcus pneumoniae*, Streptococcus pseudopneumoniae]	[Streptococcus mitis, Streptococcus pneumoniae*]	99.79
29741	Bacteroides thetaiotaomicron	Bacteroides thetaiotaomicron	[Bacteroides thetaiotaomicron*]	[Bacteroides thetaiotaomicron*]	99.78
43055	Eggerthella lenta	Eggerthella lenta	[Eggerthella lenta*]	[Eggerthella lenta*, Eggerthella timonensis]	99.32
51299	Enterococcus faecalis	Enterococcus faecalis	[Enterococcus faecalis*]	[Enterococcus faecalis*]	100.00
8176	Moraxella catarrhalis	Moraxella catarrhalis	[Moraxella canis, Moraxella catarrhalis*, Moraxella nonliquefaciens]	[Moraxella canis, Moraxella catarrhalis*]	100.00
BAA-1705	Klebsiella pneumoniae	Klebsiella pneumoniae	[Klebsiella variicola, Klebsiella quasivariicola*]	[Klebsiella pneumoniae subsp. rhinoscleromatis*, Klebsiella quasipneumoniae subsp. *quasipneumoniae*]	98.93
13637	Stenotrophomonas maltophilia	Stenotrophomonas maltophilia	[Stenotrophomonas maltophilia*]	[Stenotrophomonas maltophilia]	100.00

a Samples were analyzed in parallel by Sanger sequencing and with the *LORCAN* approach. The resulting consensus sequences were submitted to the online taxonomic identification platform leBIBI QBPP.

b Square brackets indicate proximal clusters. Asterisks indicate closest sequences based on patristic distances.

## RESULTS

We present a standardized laboratory workflow accompanied by a fully automated analysis pipeline, which together provide a sample-to-report solution for taxonomic identification of bacterial cultures based on amplicon sequencing of their 16S rRNA genes (Fig. 1). The laboratory workflow, which was tested and adjusted for parallel processing of up to 16 samples done manually by a single person (theoretically scalable up to 96 samples using automation), includes stringent quality control steps to guarantee consistent results. The whole procedure has been running under ISO/IEC 17025 accreditation standards since January 2019 in our microbial diagnostic department. The analysis pipeline is based on publicly available software components and runs on Linux servers or workstations. It automates quality control, demultiplexing, consensus sequence generation, taxonomic analysis based on the highly curated leBIBI 16S database, as well as report generation (text, PDF; see example report in the supplemental material). Turnaround time from raw amplicons to PDF reporting is about 8 h (consisting of 6 h wet lab, 1 h sequencing, and 1 h bioinformatic analysis). Validation of the sequencing results was conducted by direct comparison to Sanger sequencing with real clinical samples consisting of pure or mixed rRNA amplicons belonging to several bacterial genera (*Bacteroides*, *Eggerthella*, *Enterococcus*, *Klebsiella*, *Mycobacterium*, *Campylobacter*, *Pseudomonas*) of expected amplicon sizes of 500 bp (longer amplicons of ca. 900 bp were also successfully analyzed with the proposed pipeline; data not shown). Additionally, we created artificial amplicon and read mixtures from different bacterial species to assess the workflow’s performance and robustness when confronted with contaminated samples. We demonstrated that by combining ONT sequencing and *LORCAN*, the accuracy of Sanger sequencing can be closely matched (>99.6% sequence identity on average) and that mixed samples can be resolved at the single-base resolution level.

### Validation of SNV discrimination and analysis of mixed samples.

**FIG 2:**
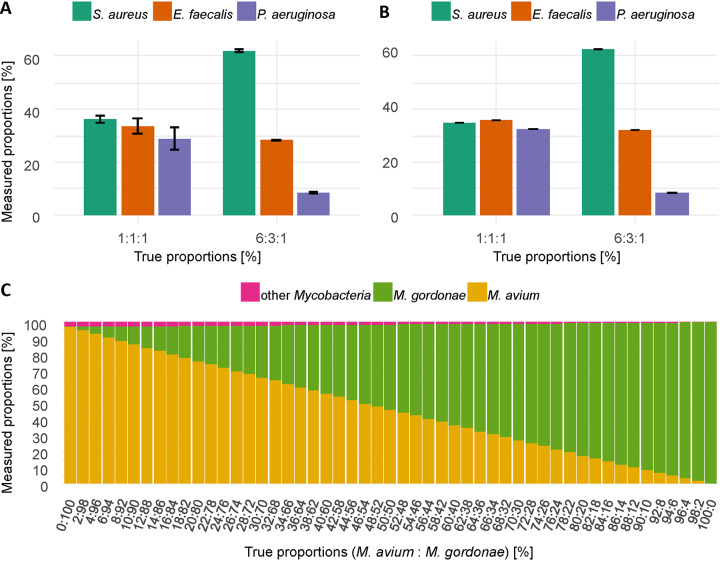
Taxonomic analysis of amplicon mixtures by *LORCAN*. Amplicons from Staphylococcus aureus, Enterococcus faecalis, and Pseudomonas aeruginosa mixed after PCR amplification (A) and mixed *in silico* from reads obtained from pure amplicons (B). Standard deviations indicate the variability across three independent replicate samples. None of the observed ratios was significantly different from the expected ratios (chi-square test for expected probabilities; *P > *0.99). (C) *In silico* mixtures of Mycobacterium gordonae and Mycobacterium avium.

To test the ability of *LORCAN* to resolve mixed samples, artificial mixtures were created by mixing either amplicons (Fig. 2A) or reads produced from pure samples (Fig. 2B and C; see also Fig. S1 and S2 in the supplemental material). The taxonomic identity of all involved strains was successfully recovered by *LORCAN*. The slightly lower amplicon length of Pseudomonas aeruginosa compared to those of Staphylococcus aureus and Enterococcus faecalis resulted in a slight underrepresentation of the latter in the mixtures (Fig. 2B) due to the narrow size window chosen for read size selection (the lower boundary of the size window around the modal read length is adjustable in the *LORCAN* command line). The mixture of two *Mycobacterium* species (97.6% sequence identity in the amplified region) (Fig. 2C) was accurately reproduced.

### Influence of database completeness on consensus accuracy and taxonomic classification.

**FIG 3:**
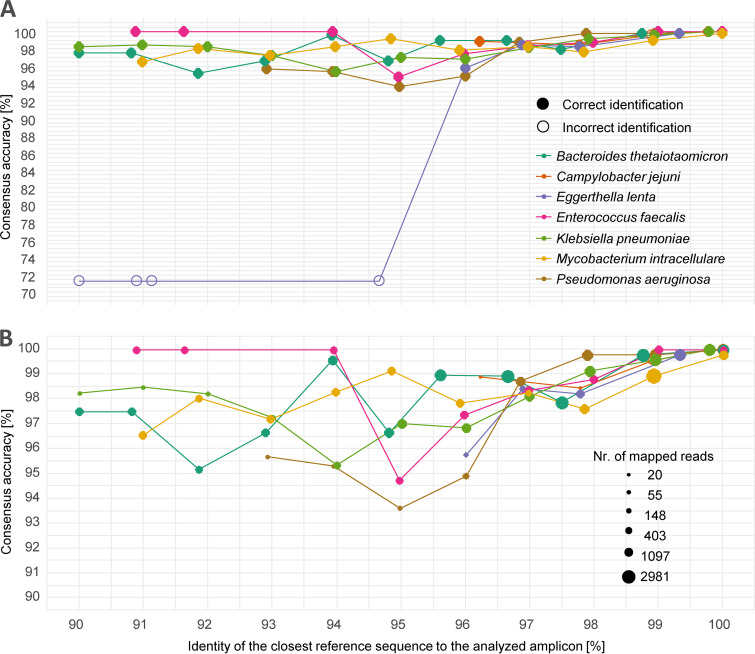
Influence of reference database completeness on consensus sequence accuracy. Each consensus sequence was compared to a consensus sequence produced with a perfectly matching reference sequence. Additionally, each consensus sequence was identified by BLAST similarity search against the full reference database. The uneven spacing of the data points reflects the database composition after subsetting. Missing values are a result of insufficient numbers of reads mapping to the reference database. (A) Filled circles indicate correct taxonomic identification of the ATCC strains. The low identities and unsuccessful identification of Eggerthella lenta are a result of a low-level contamination in combination with unsuccessful mapping of the *Eggerthella* reads. (B) The diameter of the circles is proportional to the number of reads mapped and further used in the consensus generation step (obtained from the *LORCAN* output). Additional detail is provided in Table S3 and Fig. S10 in the supplemental material.

We analyzed the influence of reference database completeness on the resulting consensus quality and accuracy by creating incomplete reference databases, from which we excluded reference sequences if they were too close to the ideal reference sequence, and then performed *LORCAN* analysis with each of these truncated databases in turn. The genetic distances of the closest reference sequences in the reference database strongly influenced the accuracy of the resulting consensus sequences. For instance, Enterococcus faecalis showed the lowest consensus accuracy at 95% database identity (Fig. 3). This was caused by gaps in the closest reference sequence available. For databases with closest identities of ≤94%, the reference sequence with the identified gaps was absent and consensus quality increased again (see Fig. S3 and S4 in the supplemental material). Classification at the species level was, however, virtually unaffected in pure amplicons. The Eggerthella lenta data set contained a contamination of Pseudomonas stutzeri reads (0.8% of all reads), which did not influence classification when reference sequences that enabled the mapping of Eggerthella lenta reads were available. In the absence of sufficiently close reference sequences, the sample was misidentified (Fig. 3A). Information provided in the *LORCAN* report did, however, reveal that the Pseudomonas stutzeri consensus sequence was only based on 20 out of 850 reads, which therefore indicated a likely case of suboptimal taxonomic classification.

### Validation of sequence consensuses generated by the combination of nanopore sequencing and *LORCAN*.

The comparison of 78 *LORCAN*-generated consensus sequences from 14 sequencing runs (including 49 clinical samples and 15 ATCC reference strains) to their corresponding Sanger sequences revealed an average sequence identity of 99.6% ± 0.6 (standard deviation). The positive control (originating from the same pool of amplicons) that was systematically sequenced in these 14 runs showed an average identity of 99.8% ± 0.2 to its corresponding Sanger sequence. All reference strains were correctly identified at the species level by *LORCAN*. Identification by leBIBI QBPP resulted in assignment of the expected species (lowest patristic distance) or the placement of the expected species in the proximal cluster of the query sequence (in the phylogenetic tree) in all but two cases. In these cases, the analyzed strains were placed in close neighborhood of the expected species in the phylogenetic tree produced by leBIBI QBPP (Table 1; see also Fig. S5 in the supplemental material).

### Comparison of sequencing costs.

**FIG 4:**
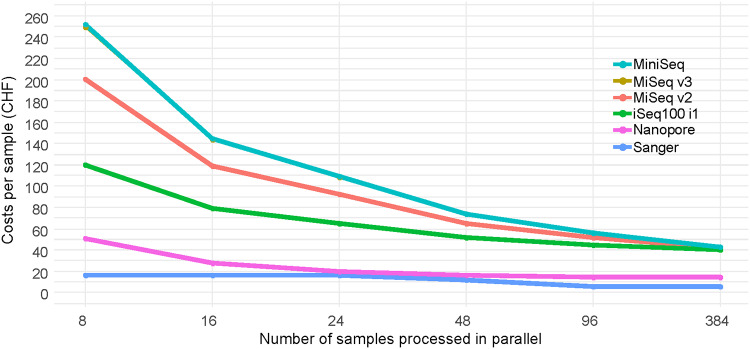
Cost estimate based on current list prices in Switzerland (currency CHF, December 2019). Prices for Illumina and Nanopore sequencing include reagents and consumables; prices for Sanger sequencing correspond to the rates at a large local service provider. The lines of MiniSeq and MiSeq v3 are confounded in the figure. Detail is provided in Table S4 in the supplemental material.

Costs per sample of the Sanger method were the lowest across different sequencing technologies (Fig. 4), provided the analyzed amplicons are pure and short enough to be covered by a single sequence at sufficient quality. Among the analyzed next-generation sequencing (NGS) methods, nanopore sequencing was by far the most cost-effective option particularly at throughputs of 24 to 48 samples. The high costs per sample for Illumina are mainly caused by the nonreusable sequencing cartridges (the full costs apply regardless of the number of processed samples) and the comparably high prices of the library preparation kits.

### Effects of parameter modifications on *LORCAN* results.

We studied the influence of the read size fraction (relative to the modal read length) and the number of input reads on *LORCAN* consensus quality. In short, optimal results were obtained when reads shorter than 20 bases below the modal read length were excluded from the analysis (see Fig. S6 in the supplemental material). Further, we found 100 reads to be sufficient for the generation of high-quality consensus sequences (see Fig. S7, S8, and S9 in the supplemental material). The required number of input reads may vary with the taxonomic complexity of the analyzed samples and the resolution required by the operator. From a theoretical viewpoint (Fig. 1B, step 2), a total of 3,000 size-selected reads may allow for the creation of high-quality consensus sequences and reliable species identification for species contributing ≥3.3% of those 3,000 selected reads (i.e., when setting a minimum reference mapping depth of 100 reads in *LORCAN*, which corresponds to the minimum number of reads recommended for reliable consensus creation [see Fig. S7]). In most cases, however, even when a sample may consist of amplicons derived from a unique species, not all reads are assigned to the target species (e.g., due to read errors and/or the presence of highly similar sequences associated with other species in the reference database). Furthermore, demultiplexing and size selection could result in significant reduction of available reads. For illustrative purposes, during our last 11 sequencing runs consisting of 89 samples (including place-holder samples; see Library preparation in Materials and Methods), an average of 639,944 ± 267,704 basecalled reads were produced, while multiplexing on average 8 ± 3 barcoded samples per sequencing run. Read demultiplexing produced thereafter an average of 46,571 ± 22,129 reads per library (i.e., in 58% of all reads, both index sequences have been identified and assigned to the same barcode). This comparably high read loss resulted from the stringent demultiplexing parameters used (detection of both 5′ and 3′ barcodes required, exclusion of reads with internal barcodes), which may effectively prevent cross talk between libraries (32). Subsequent size selection (read length of −20 to +100 bp around the modal sequence length) resulted in an average of 43,265 ± 21,305 reads per barcode, which were available for further processing. Samples producing more than 3,000 reads of the expected amplicon size were further down-sampled (adjustable *LORCAN* parameter), resulting in an average number of used reads of 3,008 ± 6 reads per sample. All samples, controls, and place holders processed in these 11 sequencing runs were successfully taxonomically identified. Although species identification could have been achieved with a lower number of reads per sample, sequence production was fast (i.e., approximately 1 to 2 h for 1 million reads), and even if flow cells may have been reused up to four times, the maximal sequencing capacity of the flow cells was never utilized (see Table S2 in the supplemental material).

## DISCUSSION

We present here the first sample-to-report solution for marker-gene-based taxonomic identification of bacterial cultures specifically designed for clinical applications. We extensively tested the influences of various analysis parameters and, therefore, provide a basis for optimal tuning of the *LORCAN* pipeline to specific requirements. We demonstrated that reads significantly shorter than the modal read length showed reduced mapability to reference sequences and that resulting consensus sequences were of reduced quality. No such observations were made when using reads from longer-length fractions (see Fig. S6 in the supplemental material). Therefore, we excluded reads that were significantly shorter than the mode of the read length distribution (by 20 bases) from the analysis with the corresponding command line parameter in *LORCAN*. With these parameters being set this way, accurate consensus sequences (≥99% identity to Sanger sequences produced from the same DNA) were reliably produced with as few as 100 size-filtered reads per sample (see Fig. S7 in the supplemental material), confirming previous findings (33).

Applicability to samples consisting of mixed amplicons was a key requirement during development of *LORCAN*, as contaminations are not rare in bacterial cultures derived from clinical samples. To exclude sources of variation due to fluctuations in wet laboratory processes, we analyzed artificially mixed amplicons based on pure reads generated from pure amplicons. *LORCAN* showed high robustness against such mixture events and was capable of quantitatively representing read compositions in mixed samples as long as the analyzed gene region and the used database provide the required taxonomic resolution. Nevertheless, we consider our approach as semiquantitative, as biases inherent to DNA extraction and amplicon generation might occur. In addition, the presence of near-identical reference sequences belonging to different species can result in elevated levels of background due to misassignment of a fraction of the reads. Although we could observe a likely bias due to this phenomenon (see Fig. S1 in the supplemental material), the bias did not prevent the correct taxonomic identification of the most abundant species in any of our experiments. Furthermore, this bias can be mitigated by choosing longer amplicons, and the planned improvement in read quality by ONT will likely improve discrimination under such conditions.

A number of studies on ONT-based marker gene analysis have been published over the past years, covering a range of different laboratory and computational approaches aiming to obtain high-quality sequences from ONT reads. Most computational workflows either include reference-based consensus generation or *de novo* assembly in combination with additional error correction steps. They were reported to perform similarly in terms of the accuracy of the produced sequences (12, 13, 15, 17, 33). *De novo* approaches are preferable when reference sequences are missing; however, so far, the only studies demonstrating “reference-free” consensus generation from complex samples (e.g., mock communities) relied on rather laborious wet lab procedures, such as rolling cycle amplification or unique tagging of the individual amplicons before sequencing (15, 16). Unlike previous studies, we specifically designed our workflow for clinical routine applications. Compatibility with mixed samples and time/cost efficacy were therefore key requirements, and comprehensive reference databases were readily available. We therefore chose a reference-based approach allowing us to separate reads originating from mixed cultures while using standard ONT protocols. Furthermore, and in contrast to most previous studies, we omitted consensus error correction, which is commonly applied to remove homopolymer errors from consensus sequences and assemblies produced from nanopore reads (12, 13), because we did not detect a negative influence of the latter errors in our taxonomic classification approach.

The strengths of our approach are that overall the procedure is faster, more flexible, and more cost-effective than Sanger or Illumina-based approaches, as it relies on both straightforward ONT protocols and automated sample analysis up to result reporting. In addition, nanopore sequencing is compatible with any amplicon size, which is a clear advantage over other existing sequencing technologies and also allows the processing and resolution of mixed amplicon samples as demonstrated here. Finally, even when the reference sequence database is incomplete or lacks closely related reference sequences, we showed that the approach is robust and provides correct taxonomic identification of the bacterial species.

Our approach has several limitations. (i) The taxonomic resolution is inherently limited by the choice of a single-gene-based approach. Commonly used 16S rRNA gene regions, for example, have been reported to allow for genus identification in >90% of cases, for species identification in 65% to 83% of cases and to result in unsuccessful identification in 1% to 14% of all analyzed isolates (8, 34, 35). Other approaches, such as matrix-assisted laser desorption ionization–time of flight (MALDI-TOF) mass spectrometry may complementarily provide fast and reliable identification of clinically relevant microorganisms (36). Yet, MALDI-TOF may also suffer from suboptimal identification due to limitations, including insufficient representation of reference species profiles in available commercial databases, absence of newly discovered species, and the existence of several commercial systems (37–39). (ii) The dependency on database quality and completeness in the *LORCAN* reference-based approach for consensus building was explored extensively by using modified databases, which lacked reference sequences closely related to the analyzed strains. Not surprisingly, consensus accuracy was strongly affected, and *LORCAN* required reference databases of high quality and completeness to reliably reach sequence qualities on par with the quality obtained by the Sanger method. Even if databases contained sequences with up to 99% identity to the analyzed species, further improvements could often be made by adding closer reference sequences (Fig. 3). Importantly though for clinical diagnostics, taxonomic identification based on the produced consensus sequence was far less affected by database completeness. Even consensus sequences produced with distant reference sequences (≤90% identity to the query sequence using an incomplete database) allowed for reliable bacterial species identification when the generated consensus was compared to a complete database. This finding indicates a high reliability of the taxonomic identification despite the database dependency of the approach. This was confirmed by extensive validation in our diagnostics department, which was based on the parallel sequencing and analysis of clinical samples using both Sanger and nanopore sequencing over several months, which overall showed average sequence identities of 99.6% (and 99.8% for positive controls sequenced conjointly with the clinical samples). (iii) Finally, the wet laboratory procedures still take several hours and would need to be optimized to allow fast and efficient processing of several samples via automation or via simplified steps.

In conclusion, we demonstrate that the combination of nanopore sequencing and *LORCAN* pipeline offers a significant improvement over the well-established Sanger or short-read sequencing approaches in terms of reliability (robustness against contaminated samples) and flexibility (read length limited by PCR only), while offering comparable turnaround time, cost, and reproducibility of the results. The described workflow has great potential to be successfully introduced in the routine of diagnostic departments and may thus facilitate custom amplicon sequencing and further taxonomic identification of bacterial pathogens.

## Supplementary Material

Supplemental file 1

Supplemental file 2

Supplemental file 3

Supplemental file 4

Supplemental file 5
