# Supplementary material for: A Sample-to-Report Solution for Taxonomic Identification of Cultured Bacteria in the Clinical Setting Based on Nanopore Sequencing
Source: J Clin Microbiol. 2020 May 26;58(6):e00060-20. doi: 10.1128/JCM.00060-20 (PMC7269405; doi:10.1128/JCM.00060-20)
Supplement: Supplemental file 1 [file JCM.00060-20-s0001.pdf]

>Enterococcus_faecalis
TGGCGGCGTGCCTAATACATGCAAGTCGAACGCTTCTTTCCTCCCGAGTGCTTGCACTCAATTGGAAAGAGGAGTGGCG
GACGGGTGAGTAACACGTGGGTAACCTACCCATCAGAGGGGGATAACACTTGGAAACAGGTGCTAATACCGCATAACAG
TTTATGCCGCATGGCATAAGAGTGAAAGGCGCTTTCGGGTGTCGCTGATGGATGGACCCGCGGTGCATTAGCTAGTTGG
TGAGGTAACGGCTCACCAAGGCCACGATGCATAGCCGACCTGAGAGGGTGATCGGCCACACTGGGACTGAGACACGGCC
CAGACTCCTACGGGAGGCAGCAGTAGGGAATCTTCGGCAATGGACGAAAGTCTGACCGAGCAACGCCGCGTGAGTGAAG
AAGGTTTTCGGATCGTAAAACTCTGTTGTTAGAGAAGAACAAGGACGTTAGTAACTGAACGTCCCCTGACGGTATCTAA
CCAGAAAGCCACGG
>Bacteroides_thetaiotaomicron
ATGCAAGTCGAGGGGCAGCATTTCAGTTTGCTTGCAAACTGGAGATGGCGACCGGCGCACGGGTGAGTAACACGTATCC
AACCTGCCGATAACTCGGGGATAGCCTTTCGAAAGAAAGATTAATACCCGATGGCATAATTAGACCGCATGGTCTTATT
ATTAAAGAATTTCGGTTATCGATGGGGATGCGTTCCATTAGGCAGTTGGTGAGGTAACGGCTCACCAAACCTTCGATGG
ATAGGGGTTCTGAGAGGAAGGTCCCCCACATTGGAACTGAGACACGGTCCAAACTCCTACGGGAGGCAGCAGTGAGGAA
TATTGGTCAATGGGCGCAGGCCTGAACCAGCCAAGTAGCGTGAAGGATGACTGCCCTATGGGTTGTAAACTTCTTTTAT
ATGGGAATAAAGTTTTCCACGTGTGGAAWTTTGTATGTACCATATGAATAAGGATCGG
>Eggerthia_lenta
TGGCGGCGTGCCTAACACATGCAAGTCGAACGATGAAACCGCCCTCGGGCGGACATGAAGTGGCGAACGGGTGAGTAAC
ACGTGACCAACCTGCCCCTTGCTCCGGGACAACCTTGGGAAACCGAGGCTAATACCGGATACTCCTCGCCCCCCTCCTG
GGGGGCCCGGGAAAGCCCAGACGGCAAGGGATGGGGTCGCGGCCCATTAGGTAGTAGGCGGGGTAACGGCCCACCTAGC
CCGCGATGGGTAGCCGGGTTGAGAGACCGACCGGCCACATTGGGACTGAGATACGGCCCAGACTCCTACGGGAGGCAGC
AGTGGGGAATTTTGCGCAATGGGGGAAACCCTGACGCAGCAACGCCGCGTGCGGGACGACGGCCTTCGGGTTGTAAACC
GCTTTCAGCAGGGAAGAAATTCGACGGTACCTGCAGAAGAAGCTCCGG
>Haemophilus_influenzae
TGGCGGCAGGCTTAACACATGCAAGTTCGAACGGTAGCAGGAGAAAGCTTGCTTTTTCTTGCTGACGAGTGGCGGACGG
GTGAGTAATGCTTGGGAATCTGGCTTATGGAGGGGGATAACGACGGGAAACTGTCGCTAATACCGCGTATTATCGGAAG
ATGAAAGTGCGGGACTGAGAGGCCGCATGCCATAGGATGAGCCCAAGTGGGATTAGGTAGTTGGTGGGGTAAAGGCCTA
CCAAGCCTGCGATCTCTAGCTGGTCTGAGAGGATGACCAGCCACACTGGAACTGAGACACGGTCCAGACTCCTACGGGA
GGCAGCAGTGGGGAATATTGCGCAATGGGGGGAACCCTGACGCAGCCATGCCGCGTGAATGAAGAAGGCCTTAGGGTTG
TAAAGTTCTTTCGGTATTGAGGAAGGTTGATGTGTTAATAGCACATCAAATTGACGTTAAATACAGAAGAAGCACCGG
>Neisseria_gonorrhoeae
TGGCGGCATGCTTTACACATGCAAGTCGGACGGCAGCACAGGGAAGCTTGCTTCTCGGGTGGCGAGTGGCGAACGGGTG
AGTAACATATCGGAACGTACCGGGTAGCGGGGGATAACTGATCGAAAGATCAGCTAATACCGCATACGTCTTGAGAGGG
AAAGCAGGGGACCTTCGGGCCTTGCGCTATCCGAGCGGCCGATATCTGATTAGCTGGTTGGCGGGGTAAAGGCCCACCA
AGGCGACGATCAGTAGCGGGTCTGAGAGGATGATCCGCCACACTGGGACTGAGACACGGCCCAGACTCCTACGGGAGGC
AGCAGTGGGGAATTTTGGACAATGGGCGCAAGCCTGATCCAGCCATGCCGCGTGTCTGAAGAAGGCCTTCGGGTTGTAA
AGGACTTTTGTCAGGGAAGAAAAGGCCGTTGCCAATATCGGCGGCCGATGACGGTACCTGAAGAATAAGCACCGG
>Pseudomonas_aeruginosa
TGGCGGCAGGCCTAACACATGCAAGTCGAGCGGATGAAGGGAGCTTGCTCCTGGATTCAGCGGCGGACGGGTGAGTAAT
GCCTAGGAATCTGCCTGGTAGTGGGGGATAACGTCCGGAAACGGGCGCTAATACCGCATACGTCCTGAGGGAGAAAGTG
GGGGATCTTCGGACCTCACGCTATCAGATGAGCCTAGGTCGGATTAGCTAGTTGGTGGGGTAAAGGCCTACCAAGGCGA
CGATCCGTAACTGGTCTGAGAGGATGATCAGTCACACTGGAACTGAGACACGGTCCAGACTCCTACGGGAGGCAGCAGT
GGGGAATATTGGACAATGGGCGAAAGCCTGATCCAGCCATGCCGCGTGTGTGAAGAAGGTCTTCGGATTGTAAAGCACT
TTAAGTTGGGAGGAAGGGCAGTAAGTTAATACCTTGCTGTTTTGACGTTACCAACAGAATAAGCACCGG
>Staphylococcus_aureus
TGGCGGCGTGCCTAATACATGCAAGTCGAGCGAACGGACGAGAAGCTTGCTTCTCTGATGTTAGCGGCGGACGGGTGAG
TAACACGTGGATAACCTACCTATAAGACTGGGATAACTTCGGGAAACCGGAGCTAATACCGGATAATATTTTGAACCGC
ATGGTTCAAAAGTGAAAGACGGTCTTGCTGTCACTTATAGATGGATCCGCGCTGCATTAGCTAGTTGGTAAGGTAACGG
CTTACCAAGGCAACGATGCATAGCCGACCTGAGAGGGTGATCGGCCACACTGGAACTGAGACACGGTCCAGACTCCTAC
GGGAGGCAGCAGTAGGGAATCTTCCGCAATGGGCGAAAGCCTGACGGAGCAACGCCGCGTGAGTGATGAAGGTCTTCGG
ATCGTAAAACTCTGTTATTAGGGAAGAACATATGTGTAAGTAACTGTGCACATCTTGACGGTACCTAATCAGAAAGCCA
CGG
>Streptococcus_pneumoniae
TGGCGGCGTGCCTAATACATGCAAGTAGAACGCTGAAGGAGGAGCTTGCTTCTCTGGATGAGTTGCGAACGGGTGAGTA
ACGCGTAGGTAACCTGCCTGGTAGCGGGGGATAACTATTGGAAACGATAGCTAATACCGCATAAGAGTAGATGTTGCAT
GACATTTGCTTAAAAGGTGCACTTGCATCACTACCAGATGGACCTGCGTTGTATTAGCTAGTTGGTGGGGTAACGGCTC
ACCAAGGCGACGATACATAGCCGACCTGAGAGGGTGATCGGCCACACTGGGACTGAGACACGGCCCAGACTCCTACGGG
AGGCAGCAGTAGGGAATCTTCGGCAATGGACGGAAGTCTGACCGAGCAACGCCGCGTGAGTGAAGAAGGTTTTCGGATC
GTAAAGCTCTGTTGTAAGAGAAGAACGAGTGTGAGAGTGGAAAGTTCACACTGTGACGGTATCTTACCAGAAAGGGACG
G
>Enterococcus_faecalis
TGGCGGCGTGCCTAATACATGCAAGTCGAACGCTTCTTTCCTCCCGAGTGCTTGCACTCAATTGGAAAGAGGAGTGGCG
GACGGGTGAGTAACACGTGGGTAACCTACCCATCAGAGGGGGATAACACTTGGAAACAGGTGCTAATACCGCATAACAG
TTTATGCCGCATGGCATAAGAGTGAAAGGCGCTTTCGGGTGTCGCTGATGGATGGACCCGCGGTGCATTAGCTAGTTGG
TGAGGTAACGGCTCACCAAGGCCACGATGCATAGCCGACCTGAGAGGGTGATCGGCCACACTGGGACTGAGACACGGCC
CAGACTCCTACGGGAGGCAGCAGTAGGGAATCTTCGGCAATGGACGAAAGTCTGACCGAGCAACGCCGCGTGAGTGAAG
AAGGTTTTCGGATCGTAAAACTCTGTTGTTAGAGAAGAACAAGGACGTTAGTAACTGAACGTCCCCTGACGGTATCTAA
CCAGAAAGCCACGG
>Helicobacter pylori
TGGCGGCGTGCCTAATACATGCAAGTCGAACGATGAAGCTTCTAGCTTGCTAGAGTGCTGATTAGTGGCGCACGGGTGA
GTAACGCATAGGTCATGTGCCTCTTAGTTTGGGATAGCCATTGGAAACGATGATTAATACCAGATACTCCCTACGGGGG
AAAGATTTATCGCTAAGAGATCAGCCTATGTCCTATCAGCTTGTTGGTAAGGTAATGGCTTACCAAGGCTATGACGGGT
ATCCGGCCTGAGAGGGTGAACGGACACACTGGAACTGAGACACGGTCCAGACTCCTACGGGAGGCAGCAGTAGGGAATA
TTGCTCAATGGGGGAAACCCTGAAGCAGCAACGCCGCGTGGAGGATGAAGGTTTTAGGATTGTAAACTCCTTTTGTTAG
AGAAGATAATGACGGTATCTAACGAATAAGCACCGG
>Campylobacter_jejuni_subsp._jejuni
TGGCGGCGTGCCTAATACATGCAAGTCGAACGATGAAGCTTCTAGCTTGCTAGAAGTGGATTAGTGGCGCACGGGTGAG
TAAGGTATAGTTAATCTGCCCTACACAAGAGGACAACAGTTGGAAACGACTGCTAATACTCTATACTCCTGCTTAACAC

AAGTTGAGTAGGGAAAGTTTTTCGGTGTAGGATGAGACTATATAGTATCAGCTAGTTGGTAAGGTAATGGCTTACCAAG
GCTATGACGCTTAACTGGTCTGAGAGGATGATCAGTCACACTGGAACTGAGACACGGTCCAGACTCCTACGGGAGGCAG
CAGTAGGGAATATTGCGCAATGGGGGAAACCCTGACGCAGCAACGCCGCGTGGAGGATGACACTTTTCGGAGCGTAAAC
TCCTTTTCTTAGGGAAGAATTCTGACGGTACCTAAGGAATAAGCACCGG
>Moraxella_catharrhalis
TGGCGGCAGGCTTAACACATGCAAGTCGAACGAAGTTAGGAAGCTTGCTTCTGATACTTAGTGGCGGACGGGTGAGTAA
TGCTTAGGAATCTGCCTAGTAGTGGGGGATAACTTGGGGAAACCCAAGCTAATACCGCATACGACCTACGGGTGAAAGG
GGGCTTTTAGCTCTCGCTATTAGATGAGCCTAAGTCGGATTAGCTGGTTGGTGGGGTAAAGGCCTACCAAGGCGACGAT
CTGTAGCTGGTCTGAGAGGATGATCAGCCACACTGGGACTGAGACACGGCCCAGACTCCTACGGGAGGCAGCAGTGGGG
AATATTGGACAATGGGCGAAAGCCTGATCCAGCCATGCCGCGTGTGTGAAGAAGGCCTTTTGGTTGTAAAGCACTTTAA
GTGGGGAGGAAAAGCTTATGGTTAATACCCATAAGCCCTGACGTTACCCACAGAATAAGCACCGG
>Klebsiella_pneumoniae
TGGCGGCAGGCCTAACACATGCAAGTCGAGCGGTAGCACAGAGAGCTTGCTCTCGGGTGACGAGCGGCGGACGGGTGAG
TAATGTCTGGGAAACTGCCTGATGGAGGGGGATAACTACTGGAAACGGTAGCTAATACCGCATAACGTCGCAAGACCAA
AGTGGGGGACCTTCGGGCCTCATGCCATCAGATGTGCCCAGATGGGATTAGCTAGTAGGTGGGGTAACGGCTCACCTAG
GCGACGATCCCTAGCTGGTCTGAGAGGATGACCAGCCACACTGGAACTGAGACACGGTCCAGACTCCTACGGGAGGCAG
CAGTGGGGAATATTGCACAATGGGCGCAAGCCTGATGCAGCCATGCCGCGTGTGTGAAGAAGGCCTTCGGGTTGTAAAG
CACTTTCAGCGGGGAGGAAGGCGTTAAGGTTAATAACCTYGTCGATTGACGTTACCCGCAGAAGAAGCACCGG
>Stenotrophomonas_maltophilia
TGGCGGTAGGCCTAACACATGCAAGTCGAACGGCAGCACAGGAGAGCTTGCTCTCTGGGTGGCGAGTGGCGGACGGGTG
AGGAATACATCGGAATCTACTTTTTCGTGGGGGATAACGTAGGGAAACTTACGCTAATACCGCATACGACCTACGGGTG
AAAGCAGGGGATCTTCGGACCTTGCGCGATTGAATGAGCCGATGTCGGATTAGCTAGTTGGCGGGGTAAAGGCCCACCA
AGGCGACGATCCGTAGCTGGTCTGAGAGGATGATCAGCCACACTGGAACTGAGACACGGTCCAGACTCCTACGGGAGGC
AGCAGTGGGGAATATTGGACAATGGGCGCAAGCCTGATCCAGCCATACCGCGTGGGTGAAGAAGGCCTTCGGGTTGTAA
AGCCCTTTTGTTGGGAAAGAAATCCAGCTGGTTAATACCCGGTTGGGATGACGGTACCCAAAGAATAAGCACCGG
>Escherichia_coli
TGGCGGCAGGCCTAACACATGCAAGTCGAACGGTAACAGAAGCAGCTTGCTGCTTTGCTGACGAGTGGCGGACGGGTGA
GTAATGTCTGGGAAACTGCCTGATGGAGGGGGATAACTACTGGAAACGGTAGCTAATACCGCATAACGTCGCAAGACCA
AAGAGGGGGACCTTCGGGCCTCTTGCCATCGGATGTGCCCAGATGGGATTAGCTAGTAGGTGGGGTAAAGGCTCACCTA
GGCGACGATCCCTAGCTGGTCTGAGAGGATGACCAGCCACACTGGAACTGAGACACGGTCCAGACTCCTACGGGAGGCA
GCAGTGGGGAATATTGCACAATGGGCGCAAGCCTGATGCAGCCATGCCGCGTGTATGAAGAAGGCCTTCGGGTTGTAAA
GTACTTTCAGCGGGGAGGAAGGGAGTAAAGTTAATACCTTTGCTCATTGACGTTACCCGCAGAAGAAGCACCGG
