# Supplementary material for: A Sample-to-Report Solution for Taxonomic Identification of Cultured Bacteria in the Clinical Setting Based on Nanopore Sequencing
Source: J Clin Microbiol. 2020 May 26;58(6):e00060-20. doi: 10.1128/JCM.00060-20 (PMC7269405; doi:10.1128/JCM.00060-20)
Supplement: Supplemental file 3 [file JCM.00060-20-s0003.pdf]

```
================== !!!! NON ACCREDITED !!!! =====================
=================================================================
***         REPORT OF SEQUENCED PCR AMPLICONS (NANOPORE)       ***
=================================================================
Analysis of barcoded sample (BC16): PosControl
Date of report: Mon Mar  4 09:29:47 2019
=================================================================
Total number of reads in the input fasta file: *662*
The reference database *BiBi16SLong* contains *69566* sequences
== A) Read counts ===================================================
Total: 664 reads aligned to references,
100.0% (664 reads) were kept after applying
  >100 cutoff reads mapping per taxonomic level

== B) Selection of taxonomic groups based on mapped reads ============
 -(515,77.6%)  Mycobacterium_intracellulare

== C) STATISTICS ABOUT CONSENSUS SEQUENCES ===========================

 Len   N   -  Taxonomic levels
----------------------------------------
 482   1   1  Mycobacterium_intracellulare
----------------------------------------
[Len]: length (bp) of the consensus sequences; [N] number of unknown bases,
i.e. not unique A/T/G/C; [-]: number of bases present in the reference sequence
but absent in the consensus sequence (deletions).
NOTE: if too many Ns are present (e.g. >3-5),
the respective consensus sequence is not of good quality.

== D) CONSENSUS SEQUENCES ============================================

== Reference group: Mycobacterium_intracellulare
GCTGGCGGCGTGCTTAACACATGCAAGTCGAACGGAAAGGCCCCTTCGGGGGTACTCGAG
TGGCGAACGGGTGAGTAACACGTGGGCAATCTGCCCTGCACTTCGGGATAAGCCTGGGAA
ACTGGGTCTAATACCGGATAGGACCTTTAGGCGCATGTCTTTAGGTGGAAAGCTTTTGCG
GTGTGGGATGGGCCCGCGGCCTATCAGCTTGTTGGTGGGGTGATGGCCTACCAAGGCGAC
GACNGGTAGCCGGCCTGAGAGGGTGTCCGGCCACACTGGGACTGAGATACGGCCCAGACT
CCTACGGGAGGCAGCAGTGGGGAATATTGCACAATGGGCGCAAGCCTGATGCAGCGACGC
CGCGTGGGGGATGACGGCCTTCGGGTTGTAAACCTCTTTCACCATCGACGAAGGTCCGGG
TTTTCTCGGATTGACGGTAGGTGGAGAAGAAGCACCGGCCAACTACGGCCAGCAGCCGCG
GT


== E) Checking the taxonomic classification of the obtained consensus sequences
(using Blastn on BiBi16SLong database)
# Query: BC16_using_Mycobacterium_intracellulare_consensus_482bp_N1_D1_Mar_04_2019
# Fields:
% id/Nber identical/alignment length/Nber mismatches/Nber gaps  subject_id
99.586/481/483/1/1 Mycobacterium_intracellulare_subsp._intracellulare~v~N~URS0000708268
99.379/480/483/2/1 Mycobacterium_chimaera~v~N~URS0000B00B0E=Bacteria-Actinobacteria-Act
99.172/479/483/3/1 Mycobacterium_arosiense~v~N~URS0000B744CF=Bacteria-Actinobacteria-Ac
99.172/479/483/3/1 Mycobacterium_intracellulare~v~N~URS0000A0C1F0=Bacteria-Actinobacter
99.168/477/481/1/3 Mycobacterium_intracellulare~v~N~URS000085F76B=Bacteria-Actinobacter
98.965/478/483/4/1 Mycobacterium_intracellulare_subsp._yongonense~v~N~URS00001E9F6A=Bac
98.758/477/483/5/1 Mycobacterium_timonense~v~N~URS000088E80D=Bacteria-Actinobacteria-Ac
98.758/477/483/5/1 Mycobacterium_colombiense~v~N~URS0000456E82=Bacteria-Actinobacteria-
98.958/475/480/2/3 Mycobacterium_intracellulare~v~N~URS00008472F8=Bacteria-Actinobacter
98.551/476/483/6/1 Mycobacterium_intracellulare~v~N~URS000080B9B8=Bacteria-Actinobacter
==================================================================
Query= BC16_using_Mycobacterium_intracellulare_consensus_482bp_N1_D1_Mar_04
_2019

Length=482
                                                                      Score     E
Sequences producing significant alignments:                          (Bits)  Value

  Mycobacterium_intracellulare_subsp._intracellulare~v~N~URS00007...  881     0.0
```

```
  Mycobacterium_chimaera~v~N~URS0000B00B0E=Bacteria-Actinobacteri...  876     0.0
  Mycobacterium_arosiense~v~N~URS0000B744CF=Bacteria-Actinobacter...  870     0.0
  Mycobacterium_intracellulare~v~N~URS0000A0C1F0=Bacteria-Actinob...  870     0.0
  Mycobacterium_intracellulare~v~N~URS000085F76B=Bacteria-Actinob...  865     0.0
  Mycobacterium_intracellulare_subsp._yongonense~v~N~URS00001E9F6...  865     0.0
  Mycobacterium_timonense~v~N~URS000088E80D=Bacteria-Actinobacter...  859     0.0
  Mycobacterium_colombiense~v~N~URS0000456E82=Bacteria-Actinobact...  859     0.0
  Mycobacterium_intracellulare~v~N~URS00008472F8=Bacteria-Actinob...  857     0.0
  Mycobacterium_intracellulare~v~N~URS000080B9B8=Bacteria-Actinob...  854     0.0


Query_1  1    GCTGGCGGCGTGCTTAACACATGCAAGTCGAACGGAAAGGCCCCTTCGGGGGTACTCGAG  60
20514    1    ............................................................  60
4412     1    ............................................................  60
17781    1    ............................................................  60
12857    1    ............................................................  60
39891    3      ..............-.......-...................................  58
14786    1    ............................................................  60
15000    1    ............................................................  60
4862     1    ..........................................T......A..........  60
43924    4       ..G..........-.......-...................................  58
65379    1    ..........................................T......A..........  60

Query_1  61   TGGCGAACGGGTGAGTAACACGTGGGCAATCTGCCCTGCACTTCGGGATAAGCCTGGGAA  120
20514    61   ............................................................  120
4412     61   ............................................................  120
17781    61   ............................................................  120
12857    61   ............................................................  120
39891    59   ............................................................  118
14786    61   ............................................................  120
15000    61   ............................................................  120
4862     61   ............................................................  120
43924    59   ............................................................  118
65379    61   ............................................................  120

Query_1  121  ACTGGGTCTAATACCGGATAGGACCTTTAGGCGCATGTCTTTAGGTGGAAAGCTTTTGCG  180
20514    121  ............................................................  180
4412     121  ............................................................  180
17781    121  ........................................A.T.................  180
12857    121  .....................................C....T.................  180
39891    119  ............................................................  178
14786    121  ..............................A...........T.................  180
15000    121  ..............................A...........T.................  180
4862     121  ..............................A...........T.................  180
43924    119  ............................................................  178
65379    121  ................A.............A...........T.................  180

Query_1  181  GTGTGGGATGGGCCCGCGGCCTATCAGCTTGTTGGTGGGGTGATGGCCTACCAAGGCGAC  240
20514    181  ............................................................  240
4412     181  ............................................................  240
17781    181  ............................................................  240
12857    181  ............................................................  240
39891    179  ............................................................  238
14786    181  ............................................................  240
15000    181  ............................................................  240
4862     181  ............................................................  240
43924    179  ............................................................  238
65379    181  ............................................................  240

Query_1  241  GACNGGTAGCCGGCCTGAGAGGGTGTCCGGCCACACTGGGACTGAGATACGGCCCAGACT  300
20514    241  ...G........................................................  300
4412     241  ...G........................................................  300
17781    241  ...G........................................................  300
12857    241  ...G........................................................  300
39891    239  ...G........................................................  298
14786    241  ...G........................................................  300
```

```
15000    241  ...G........................................................  300
4862     241  ...G........................................................  300
43924    239  ...G........................................................  298
65379    241  ...G........................................................  300

Query_1  301  CCTACGGGAGGCAGCAGTGGGGAATATTGCACAATGGGCGCAAGCCTGATGCAGCGACGC  360
20514    301  ............................................................  360
4412     301  ............................................................  360
17781    301  ............................................................  360
12857    301  ............................................................  360
39891    299  ............................................................  358
14786    301  ............................................................  360
15000    301  ............................................................  360
4862     301  ............................................................  360
43924    299  ............................................................  358
65379    301  ............................................................  360

Query_1  361  CGCGTGGGGGATGACGGCCTTCGGGTTGTAAACCTCTTTCACCATCGACGAAGGTCCGGG  420
20514    361  ............................................................  420
4412     361  .......................................................T....  420
17781    361  ............................................................  420
12857    361  ............................................................  420
39891    359  ............................................................  418
14786    361  .......................................................T....  420
15000    361  .......................................................T....  420
4862     361  ............................................................  420
43924    359  ............................................................  418
65379    361  ............................................................  420

Query_1  421  TTTTCTCGGATTGACGGTAGGTGGAGAAGAAGCACCGGCCAACTACG-GCCAGCAGCCGC  479
20514    421  ...............................................T............  480
4412     421  ...............................................T............  480
17781    421  ...............................................T............  480
12857    421  ...............................................T............  480
39891    419  ...............................................T............  478
14786    421  ...............................................T............  480
15000    421  .........C.....................................T............  480
4862     421  ...............................................T............  480
43924    419  ...............................................T............  478
65379    421  ...............................................T............  480

Query_1  480  GGT  482
20514    481  ...  483
4412     481  ...  483
17781    481  ...  483
12857    481  ...  483
39891    479  ...  481
14786    481  ...  483
15000    481  ...  483
4862     481  ...  483
43924    479  ...  481
65379    481  ...  483


====================================================================
== F) PHYLOGENETIC TREE ============================================
====================================================================
MAXIMUM LIKELIHOOD TREE
-----------------------

Log-likelihood of the tree: -745.0553 (s.e. 26.8652)
Unconstrained log-likelihood (without tree): -718.9665
Number of free parameters (#branches + #model parameters): 26
Akaike information criterion (AIC) score: 1542.1105
Corrected Akaike information criterion (AICc) score: 1545.2656
Bayesian information criterion (BIC) score: 1650.1920
```

```
Total tree length (sum of branch lengths): 0.0301
Sum of internal branch lengths: 0.0108 (35.7327% of tree length)

WARNING: 1 near-zero internal branches (<0.0021) should be treated with caution
         Such branches are denoted by '**' in the figure below

NOTE: Tree is UNROOTED although outgroup taxon 'Mycobacterium_intracellulare~v~N~URS000
Numbers in parentheses are  ultrafast bootstrap support (%)
```

```
+*******Mycobacterium_intracellulare~v~N~URS000080B9B8=Bacteria-Actinobacteria-Actinoba
|
|                               +**BC16_using_Mycobacterium_intracellulare_consensus_48
|                       +-------| (60)
|                       |       +**Mycobacterium_intracellulare_subsp._intracellulare~v
|                       |       |
|                       |       +-------Mycobacterium_chimaera~v~N~URS0000B00B0E=Bacter
|                       |       |
|                       |       |       +-------Mycobacterium_intracellulare~v~N~URS000
|                       |       +-------| (77)
|                       |               +----------------Mycobacterium_intracellulare~v
|               +-------| (61)
|               |       +-------Mycobacterium_arosiense~v~N~URS0000B744CF=Bacteria-Acti
|               |       |
|               |       +-------Mycobacterium_intracellulare~v~N~URS0000A0C1F0=Bacteria
+---------------| (93)
|               |       +**Mycobacterium_intracellulare_subsp._yongonense~v~N~URS00001E
|               +*******| (69)
|                       +*******Mycobacterium_timonense~v~N~URS000088E80D=Bacteria-Acti
|
+**Mycobacterium_intracellulare~v~N~URS000080B9B8=Bacteria-Actinobacteria-Actinobacteri

====================================================================
```
