# Supplementary material for: A Sample-to-Report Solution for Taxonomic Identification of Cultured Bacteria in the Clinical Setting Based on Nanopore Sequencing
Source: J Clin Microbiol. 2020 May 26;58(6):e00060-20. doi: 10.1128/JCM.00060-20 (PMC7269405; doi:10.1128/JCM.00060-20)
Supplement: Supplemental file 4 [file JCM.00060-20-s0004.pdf]

>BC33_using_Campylobacter_jejuni_consensus_472bp_N0_D1_Apr_08_2019
GCTGGCGGCGTGCCTAATACATGCAAGTCGAACGATGAAGCTTCTAGCTTGCTAGAAGTGGATTAGTGGCGCACGGGTGAGTAA
GGTATAGTTAATCTGCCCTACACAAGAGGACAACAGTTGGAAACGACTGCTAATACTCTATACTCCTGCTTAACACAAGTTGAGTA
GGGAAAGTTTTTCGGTGTAGGATGAGACTATATAGTATCAGCTAGTTGGTAAGGTAATGGCTTACCAAGGCTATGACGCTTAACT
GGTCTGAGAGGATGATCAGTCACACTGGAACTGAGACACGGTCCAGACTCCTACGGGAGGCAGCAGTAGGGAATATTGCGCAA
GGGGGAAACCCTGACGCAGCAACGCCGCGTGGAGGATGACACTTTTCGGAGCGTAAACTCCTTTTCTTAGGGAAGAATTCTGAC
GGTACCTAAGGAATAAGCACCGGCTAACTCCGTGCCAGCAGCCGCGGTAA
>BC34_using_Helicobacter_pylori_consensus_458bp_N0_D1_Apr_08_2019
GCTGGCGGCGTGCCTAATACATGCAAGTCGAACGATGAAGCTTCTAGCTTGCTAGAGTGCTGATTAGTGGCGCACGGGTGAGTA
ACGCATAGGTCATGTGCCTCTTAGTTTGGGATAGCCATTGGAAACGATGATTAATACCAGATACTCCCTACGGGGAAAGATTTATC
GCTAAGAGATCAGCCTATGTCCTATCAGCTTGTTGGTAAGGTAATGGCTTACCAAGGCTATGACGGGTATCCGGCCTGAGAGGG
TGAACGGACACACTGGAACTGAGACACGGTCCAGACTCCTACGGGAGGCAGCAGTAGGGAATATTGCTCAATGGGGAAACCCT
GAAGCAGCAACGCCGCGTGGAGGATGAAGGTTTTAGGATTGTAAACTCCTTTTGTTAGAGAAGATAATGACGGTATCTAACGAAT
AAGCACCGGCTAACTCCGTGCCAGCAGCCGCGGTAA
>BC35_using_Enterococcus_faecalis_consensus_519bp_N0_D1_Apr_08_2019
GCTGGCGGCGTGCCTAATACATGCAAGTCGAACGCTTCTTTCCTCCCGAGTGCTTGCACTCAATTGGAAAGAGGAGTGGCGGAC
GGGTGAGTAACACGTGGGTAACCTACCCATCAGAGGGGGATAACACTTGGAAACAGGTGCTAATACCGCATAACAGTTTATGCC
GCATGGCATAAGAGTGAAAGGCGCTTTCGGGTGTCGCTGATGGATGGACCCGCGGTGCATTAGCTAGTTGGTGAGGTAACGGC
TCACCAAGGCCACGATGCATAGCCGACCTGAGAGGGTGATCGGCCACACTGGGACTGAGACACGGCCCAGACTCCTACGGGAG
GCAGCAGTAGGGAATCTTCGGCAATGGACGAAAGTCTGACCGAGCAACGCCGCGTGAGTGAAGAAGGTTTTCGGATCGTAAAAC
TCTGTTGTTAGAGAAGAACAAGGACGTTAGTAACTGAACGTCCCCTGACGGTATCTAACCAGAAAGCCACGGCTAACTACGTGCC
AGCAGCCGCGGTAATA
>BC36_using_Escherichia_coli_consensus_500bp_N0_D0_Apr_08_2019
GCTGGCGGCAGGCCTAACACATGCAAGTCGAACGGTAACAGGAAGCAGCTTGCTGCTTTGCTGACGAGTGGCGGACGGGTGAG
TAATGTCTGGGAAACTGCCTGATGGAGGGGGATAACTACTGGAAACGGTAGCTAATACCGCATAACGTCGCAAGACCAAAGAGG
GGGACCTTCGGGCCTCTTGCCATCGGATGTGCCCAGATGGGATTAGCTAGTAGGTGGGGTAAAGGCTCACCTAGGCGACGATC
CCTAGCTGGTCTGAGAGGATGACCAGCCACACTGGAACTGAGACACGGTCCAGACTCCTACGGAGGCAGCAGTGGGGAATATT
GCACAATGGGCGCAAGCCTGATGCAGCCATGCCGCGTGTATGAAGAAGGCCTTCGGGTTGTAAAGTACTTTCAGCGGGGAGGA
AGGGAGTAAAGTTAATACCTTTGCTCATTGACGTTACCCGCAGAAGAAGCACCGGCTAACTCCGTGCCAGCAGCCGCGGTAATA
>BC37_using_Haemophilus_influenzae_consensus_497bp_N0_D2_Apr_08_2019
GCTGGCGGCAGGCTTAACACATGCAAGTCGAACGGTAGCAGGAGAAAGCTTGCTTTCTTGCTGACGAGTGGCGGACGGGTGAG
TAATGCTTGGGAATCTGGCTTATGGAGGGGGATAACGACGGGAAACTGTCGCTAATACCGCGTATTATCGGAAGATGAAAGTGC
GGGACTGAGAGGCCGCATGCCATAGGATGAGCCCAAGTGGGATTAGGTAGTTGGTGGGGTAAAGGCCTACCAAGCCTGCGATC
TCTAGCTGGTCTGAGAGGATGACCAGCCACACTGGAACTGAGACACGGTCCAGACTCCTACGGGAGGCAGCAGTGGGGAATAT
TGCGCAAGGGGGAACCCTGACGCAGCCATGCCGCGTGAATGAAGAAGGCCTTAGGGTTGTAAAGTTCTTTCGGTATTGAGGAAG
GTTGATGTGTTAATAGCACATCAAATTGACGTTAAATACAGAAGAAGCACCGGCTAACTCCGTGCCAGCAGCCGCGGTAA
>BC38_using_Neisseria_gonorrhoeae_consensus_504bp_N0_D0_Apr_08_2019
GCTGGCGGCATGCTTTACACATGCAAGTCGGACGGCAGCACAGGGAAGCTTGCTTCTCGGGTGGCGAGTGGCGAACGGGTGA
GTAACATATCGGAACGTACCGGGTAGCGGGGGATAACTGATCGAAAGATCAGCTAATACCGCATACGTCTTGAGAGGGAAAGCA
GGGGACCTTCGGGCCTTGCGCTATCCGAGCGGCCGATATCTGATTAGCTGGTTGGCGGGGTAAAGGCCCACCAAGGCGACGAT
CAGTAGCGGGTCTGAGAGGATGATCCGCCACACTGGGACTGAGACACGGCCCAGACTCCTACGGGAGGCAGCAGTGGGGAATT
TTGGACAATGGGCGCAAGCCTGATCCAGCCATGCCGCGTGTCTGAAGAAGGCCTTCGGGTTGTAAAGGACTTTTGTCAGGGAAG
AAAAGGCCGTTGCCAATATCGGCGGCCGATGACGGTACCTGAAGAATAAGCACCGGCTAACTACGTGCCAGCAGCCGCGGTAA
GACGT
>BC39_using_Pseudomonas_aeruginosa_consensus_494bp_N0_D0_Apr_08_2019
GCTGGCGGCAGGCCTAACACATGCAAGTCGAGCGGATGAAGGGAGCTTGCTCCTGGATTCAGCGGCGGACGGGTGAGTAATGC
CTAGGAATCTGCCTGGTAGTGGGGGATAACGTCCGGAAACGGGCGCTAATACCGCATACGTCCTGAGGGAGAAAGTGGGGGAT
CTTCGGACCTCACGCTATCAGATGAGCCTAGGTCGGATTAGCTAGTTGGTGGGGTAAAGGCCTACCAAGGCGACGATCCGTAAC
TGGTCTGAGAGGATGATCAGTCACACTGGAACTGAGACACGGTCCAGACTCCTACGGGAGGCAGCAGTGGGGAATATTGGACAAT
GGGCGAAAGCCTGATCCAGCCATGCCGCGTGTGTGAAGAAGGTCTTCGGATTGTAAAGCACTTTAAGTTGGGAGGAAGGGCAGT
AAGTTAATACCTTGCTGTTTTGACGTTACCAACAGAATAAGCACCGGCTAACTTCGTGCCAGCAGCCGCGGTAATA
>BC40_using_Staphylococcus_aureus_consensus_507bp_N0_D0_Apr_08_2019
GCTGGCGGCGTGCCTAATACATGCAAGTCGAGCGAACGGACGAGAAGCTTGCTTCTCTGATGTTAGCGGCGGACGGGTGAGTA
ACACGTGGATAACCTACCTATAAGACTGGGATAACTTCGGGAAACCGGAGCTAATACCGGATAATATTTTGAACCGCATGGTTCA
AAAGTGAAAGACGGTCTTGCTGTCACTTATAGATGGATCCGCGCTGCATTAGCTAGTTGGTAAGGTAACGGCTTACCAAGGCAAC
GATGCATAGCCGACCTGAGAGGGTGATCGGCCACACTGGAACTGAGACACGGTCCAGACTCCTACGGGAGGCAGCAGTAGGGA
ATCTTCCGCAATGGGCGAAAGCCTGACGGAGCAACGCCGCGTGAGTGATGAAGGTCTTCGGATCGTAAAACTCTGTTATTAGGA
AGAACATATGTGTAAGTAACTGTGCACATCTTGACGGTACCTAATCAGAAAGCCACGGCTAACTACGTGCCAGCAGCCGCGGTAA
TA
>BC41_using_Streptococcus_pneumoniae_consensus_503bp_N0_D0_Apr_08_2019
GCTGGCGGCGTGCCTAATACATGCAAGTAGAACGCTGAAGGAGGAGCTTGCTTCTCTGGATGAGTTGCGAACGGGTGAGTAAC
GCGTAGGTAACCTGCCTGGTAGCGGGGGATAACTATTGGAAACGATAGCTAATACCGCATAAGAGTAGATGTTGCATGACATTTGC
TTAAAAGGTGCACTTGCATCACTACCAGATGGACCTGCGTTGTATTAGCTAGTTGGTGGGGTAACGGCTCACCAAGGCGACGATA
CATAGCCGACCTGAGAGGGTGATCGGCCACACTGGGACTGAGACACGGCCCAGACTCCTACGGGAGGCAGCAGTAGGGAATCT
TCGGCAATGGACGGAAGTCTGACCGAGCAACGCCGCGTGAGTGAAGAAGGTTTTCGGATCGTAAAGCTCTGTTGTAAGAGAAGA
ACGAGTGTGAGAGTGGAAAGTTCACACTGTGACGGTATCTTACCAGAAAGGGACGGCTAACTACGTGCCAGCAGCCGCGGTAA
>BC42_using_Bacteroides_thetaiotaomicron_consensus_500bp_N0_D0_Apr_08_2019
GCTAGCTACAGGCTTAACACATGCAAGTCGAGGGGCAGCATTTCAGTTTGCTTGCAAACTGGAGATGGCGACCGGCGCACGGGT
GAGTAACACGTATCCAACCTGCCGATAACTCGGGGATAGCCTTTCGAAAGAAAGATTAATACCCGATGGCATAATTAGACCGCAT
GGTCTTATTATTAAAGAATTTCGGTTATCGATGGGGATGCGTTCCATTAGGCAGTTGGTGAGGTAACGGCTCACCAAACCTTCGA
TGGATAGGGGTTCTGAGAGGAAGGTCCCCCACATTGGAACTGAGACACGGTCCAAACTCCTACGGGAGGCAGCAGTGAGGAAT
ATTGGTCAATGGGCGCAGGCCTGAACCAGCCAAGTAGCGTGAAGGATGACTGCCCTATGGGTTGTAAACTTCTTTTATATGGGAA
TAAAGTTTTCCACGTGTGGAATTTTGTATGTACCATATGAATAAGGATCGGCTAACTCCGTGCCAGCAGCCGCGGTAA
>BC43_using_Eggerthella_lenta_consensus_470bp_N1_D2_Apr_08_2019
GCTGGCGGCGTGCCTAACACATGCAAGTCGAACGATGAAACCGCCCTCGGGCGGACATGAAGTGGCGAACGGGTGAGTAACAC
GTGACCAACCTGCCCCTTGCTCCGGGACAACCTTGGGAAACCGAGGCTAATACCGGATACTCCTCCCCCCCTCCTGGGGGGCC
CNGGAAAGCCCAGACGGCAAGGGATGGGGTCGCGGCCCATTAGGTAGTAGGCGGGGTAACGGCCCACCTAGCCCGCGATGGG

TAGCCGGGTTGAGAGACCGACCGGCCACATTGGGACTGAGATACGGCCCAGACTCCTACGGGAGGCAGCAGTGGGGAATTTTG
CGCAAGGGGGAAACCCTGACGCAGCAACGCCGCGTGCGGGACGACGGCCTTCGGGTTGTAAACCGCTTTCAGCAGGGAAGAA
ATTCGACGGTACCTGCAGAAGAAGCTCCGGCTAACTACGTGCCAGCAGCCGCGGTAA
>BC44_using_Enterococcus_faecalis_consensus_517bp_N0_D0_Apr_08_2019
GCTGGCGGCGTGCCTAATACATGCAAGTCGAACGCTTCTTTCCTCCCGAGTGCTTGCACTCAATTGGAAAGAGGAGTGGCGGAC
GGGTGAGTAACACGTGGGTAACCTACCCATCAGAGGGGGATAACACTTGGAAACAGGTGCTAATACCGCATAACAGTTTATGCC
GCATGGCATAAGAGTGAAAGGCGCTTTCGGGTGTCGCTGATGGATGGACCCGCGGTGCATTAGCTAGTTGGTGAGGTAACGGC
TCACCAAGGCCACGATGCATAGCCGACCTGAGAGGGTGATCGGCCACACTGGGACTGAGACACGGCCCAGACTCCTACGGGAG
GCAGCAGTAGGGAATCTTCGGCAATGGACGAAAGTCTGACCGAGCAACGCCGCGTGAGTGAAGAAGGTTTTCGGATCGTAAAAC
TCTGTTGTTAGAGAAGAACAAGGACGTTAGTAACTGAACGTCCCCTGACGGTATCTAACCAGAAAGCCACGGCTAACTACGTGCC
AGCAGCCGCGGTAA
>BC45_using_Moraxella_catarrhalis_consensus_496bp_N0_D0_Apr_08_2019
GCTGGCGGCAGGCTTAACACATGCAAGTCGAACGAAGTTAGGAAGCTTGCTTCTGATACTTAGTGGCGGACGGGTGAGTAATGC
TTAGGAATCTGCCTAGTAGTGGGGGATAACTTGGGGAAACCCAAGCTAATACCGCATACGACCTACGGGTGAAAGGGGGCTTTT
AGCTCTCGCTATTAGATGAGCCTAAGTCGGATTAGCTGGTTGGTGGGGTAAAGGCCTACCAAGGCGACGATCTGTAGCTGGTCT
GAGAGGATGATCAGCCACACTGGGACTGAGACACGGCCCAGACTCCTACGGGAGGCAGCAGTGGGGAATATTGGACAATGGGC
GAAAGCCTGATCCAGCCATGCCGCGTGTGTGAAGAAGGCCTTTTGGTTGTAAAGCACTTTAAGTGGGGAGGAAAAGCTTATGGT
TAATACCCATAAGCCCTGACGTTACCCACAGAATAAGCACCGGCTAACTCTGTGCCAGCAGCCGCGGTAAGACAGAG
>BC46_using_Klebsiella_pneumoniae_consensus_498bp_N2_D0_Apr_08_2019
GCTGGCGGCAGGCCTAACACATGCAAGTCGAGCGGTAGCACAGAGAGCTTGCTCTCGGGTGACGAGCGGCGGACGGGTGAGT
AATGTCTGGGAAACTGCCTGATGGAGGGGGATAACTACTGGAAACGGTAGCTAATACCGCATAACGTCGCAAGACCAAAGTGGGG
GACCTTCGGGCCTCATGCCATCAGATGTGCCCAGATGGGATTAGCTAGTAGGTGGGGTAACGGCTCACCTAGGCGACGATCCCT
AGCTGGTCTGAGAGGATGACCAGCCACACTGGAACTGAGACACGGTCCAGACTCCTACGGGAGGCAGCAGTGGGGAATATTGC
ACAATGGGCGCAAGCCTGATGCAGCCATGCCGCGTGTGTGAAGAAGGCCTTCGGGTTGTAAAGCACTTTCAGCGGGGAGGAAG
GCGNTNAGGTTAATAACCTCATCGATTGACGTTACCCGCAGAAGAAGCACCGGCTAACTCCGTGCCAGCAGCCGCGGTAATA
>BC47_using_Stenotrophomonas_maltophilia_consensus_499bp_N0_D0_Apr_08_2019
GCTGGCGGTAGGCCTAACACATGCAAGTCGAACGGCAGCACAGGAGAGCTTGCTCTCTGGGTGGCGAGTGGCGGACGGGTGA
GGAATACATCGGAATCTACTTTTTCGTGGGGGATAACGTAGGGAAACTTACGCTAATACCGCATACGACCTACGGGTGAAAGCAG
GGGATCTTCGGACCTTGCGCGATTGAATGAGCCGATGTCGGATTAGCTAGTTGGCGGGGTAAAGGCCCACCAAGGCGACGATC
CGTAGCTGGTCTGAGAGGATGATCAGCCACACTGGAACTGAGACACGGTCCAGACTCCTACGGGAGGCAGCAGTGGGGAATAT
TGGACAATGGGCGCAAGCCTGATCCAGCCATACCGCGTGGGTGAAGAAGGCCTTCGGGTTGTAAAGCCCTTTTGTTGGGAAAGA
AATCCAGCTGGTTAATACCCGGTTGGGATGACGGTACCCAAAGAATAAGCACCGGCTAACTTCGTGCCAGCAGCCGCGGTAA
>BC48_using_Mycobacterium_intracellulare_consensus_485bp_N0_D0_Apr_08_2019
GCTGGCGGCGTGCTTAACACATGCAAGTCGAACGGAAAGGCCCCTTCGGGGGTACTCGAGTGGCGAACGGGTGAGTAACACGT
GGGCAATCTGCCCTGCACTTCGGGATAAGCCTGGGAAACTGGGTCTAATACCGGATAGGACCTTTAGGCGCATGTCTTTAGGTG
GAAAGCTTTTGCGGTGTGGGATGGGCCCGCGGCCTATCAGCTTGTTGGTGGGGTGATGGCCTACCAAGGCGACGACGGGTAGC
CGGCCTGAGAGGGTGTCCGGCCACACTGGGACTGAGATACGGCCCAGACTCCTACGGGAGGCAGCAGTGGGGAATATTGCAC
AATGGGCGCAAGCCTGATGCAGCGACGCCGCGTGGGGGATGACGGCCTTCGGGTTGTAAACCTCTTTCACCATCGACGAAGGT
CCGGGTTTTCTCGGATTGACGGTAGGTGGAGAAGAAGCACCGGCCAACTACGTGCCAGCAGCCGCGGTAA
